# Problem gambling among secondary school adolescents in Enugu, Nigeria

**DOI:** 10.4314/ahs.v23i3.86

**Published:** 2023-09

**Authors:** Awoere T Chinawa, Edmund N Ossai, Paul C Odinka, Obinna C Nduaguba, Jaclyn I Odinka, Ann E Aronu, Josephat M Chinawa

**Affiliations:** 1 Enugu state University Teaching hospital, Enugu State; 2 Department of Community Medicine, College of Health Sciences, Ebonyi State University Abakaliki, Nigeria; 3 Department of Paediatrics, College of Medicine, University of Nigeria Enugu Campus, Nigeria; 4 Department of Psychological Medicine, University of Nigeria Enugu Campus; 5 Department of Paediatrics, Enugu State University Teaching hospital, Enugu State; 6 Social Sciences Unit, School of General Studies, University of Nigeria Nsukka

**Keywords:** Problem gambling, secondary school, adolescents, internet addiction, gambling severity index

## Abstract

**Background:**

Adolescent problem gambling is a common behavioural issue and could be associated with internet addiction.

**Objectives:**

To determine the pattern of problem gambling and factors that predict gambling among adolescents that attended secondary schools in southeast Nigeria.

**Methodology:**

This is a cross-sectional study design. A three-stage sampling technique was used to select 796 secondary school adolescents from eight secondary schools in Enugu State, Nigeria.

**Results:**

The highest proportion of the respondents, 38.3% had problem gambling with negative consequences. There was a weak positive correlation between gambling severity index and internet addiction. (n=796, r=0.254, p<0.001). There is a very weak negative correlation between gambling severity index and age in years. (n=796, r= -0.034, p=0.331).

The male respondents were 1.5 times more likely to have problem gambling when compared with the females, (AOR=1.5; (95%CI: 1.1-2.0). The respondents who have internet addiction were 3.5 times more likely to have problem gambling when compared with those who do not have an internet addiction, (AOR=3.5; 95%CI: 2.6-4.8).

**Conclusion:**

The prevalence of gambling among adolescents is high. Adolescents who had problem gambling also had internet addiction. The male respondents were more likely to have problem gambling than their female folk.

## Introduction

Gambling is simply defined as putting lots of value especially money at risk to get something of a higher and better reward. 1 Problem gambling, a gambling disorder, according to DSM-5, is defined as a repetitive and uncontrollable gambling behaviour despite harm and negative consequences. 2-5 Problem gambling is an addictive behavioural disorder with associated impulsivity. It could lead to adverse and severe social malaise.

Adolescents get involved in all types of gambling. This ranges from lottery, cards, dice, board games, sports betting, to games of personal skill. 4 Over the last decade, the Annenberg public policy center noted that monthly use of internet gambling sites among secondary school males rose by about 400% from 2008 to 2010 with an increase of about 3% every week. 4

Online gambling has increased notably in the last 6 years in Norway. Online gamblers compared to offline gamblers were more likely to be males. 4 Though female adolescents are noted to gamble less than the males, recent studies have shown increased rates of off-line gambling on sporting activities among the female adolescent child. 4 Natale 5 et al pointed-out family influence as a factor that influences adolescent gambling. They noted that adolescent gambling was five times higher among online gamblers than non-online gamblers with factors such as living with non-biological parents and higher family socioeconomic status as predictors. 5

Gambling is a behavioural disorder that can cause severe effects on the health of the adolescent child. Studies have also shown a link between gambling and certain criminal conduct. 6

Other effects of gambling among adolescents include bankruptcy, school drop-out, the use and abuse of substances, depression, para-suicide, and suicide. 7,8

Studies have shown that a combination of biological, social and psychological factors contribute to gambling behaviours. 9,10 For instance, a meta-analysis has elicited individual risk factors such as alcohol use frequency, depression, male gender, antisocial behaviours, substance use, previous gambling activities, tobacco use, violence, temperament, peer antisocial behaviour, poor academic performance as predictors of gambling among adolescents. 11 They also noted resilience and family support, female gender, emotional intelligence, social competence and family cohesion as protective factors. 12-18

Studies on gambling among adolescents are scanty in South-East Nigeria. Works on this topic were clustered in South-West Nigeria. For instance, Bankole 19 et al investigated the patterns and prevalence of gambling behaviour among youths in Oyo and Ekiti, South West Nigeria using a self-report questionnaire. They noted that the most common pattern of gambling is sports betting especially football and basketball. Moderate betting is the commonest form of gambling with a prevalence of 64.3%. Besides, Adebisi 20 et al noted gambling as a coping mechanism among adolescents aged 15 and 29 years.

Recent polls conducted in Nigeria showed rising trends of gambling and betting. One of the polls revealed that seventy-seven percent (77%) of people attested to the high prevalence of betting and gambling especially in the South-West (92 percent) and South-South (91 percent) geo-political zones. 21 It also revealed Bet9ja (64 percent), Nairabet (34 percent), Pool (22 percent) and Lotto (20 percent) as the commonest betting platforms in the country. 21

This present study is the first done in the Enugu State University Teaching Hospital. It will be very useful as reference point for gambling studies in this locale. This study is important as it drew its sample across several secondary schools in the South-East Nigeria. Furthermore, the few works cited on gambling in Nigeria are of small sample size and the impact of rural /urban influence was not well ascertained. This work will also help in raising awareness on the deleterious effects of internet/gambling addiction on adolescents.

This work is therefore aimed at determining the pattern of gambling and factors that predict gambling among adolescents that attended secondary schools in southeast Nigeria

## Methods

### Study area and population

This is a cross-sectional study that enrolled a total of seven hundred and ninety-six secondary school adolescents across six secondary schools from June 2021 to September 2021. There are a total of 87 secondary schools in Enugu State. The secondary schools enrolled in the study were representative of the population of secondary school in Enugu state at the time of the study. Adolescents who participated in the study were recruited from a frame of private and public schools. All information concerning this study and the contents of the questionnaire were explained to the students.

### Sampling technique

A three-stage sampling technique was used to select the respondents for inclusion in the study.

There are 17 local government areas (LGAs) in the state of which five are regarded as urban and twelve as rural LGAs. In the first stage, the LGAs were first stratified into urban and rural LGAs and using a simple random sampling technique of balloting, two LGAs were selected from the urban LGAs and another two from the rural LGAs. In the second stage, a list of the secondary schools in each of the selected LGAs in the urban and rural areas was made and using a simple random sampling technique of balloting, two secondary schools were selected in each of the four selected LGAs.

Each school contributed at least 90 respondents to the study. In each of the selected schools, the number of students in the senior secondary class including senior secondary 1, 2, and 3 classes was obtained and this served as the sampling frame. Dividing this number by 90, a sampling interval was obtained. The first respondent was selected using a simple random sampling technique of balloting after which the interval was applied.

### Study design

This is a cross-sectional study among adolescents attending secondary schools in Enugu State.

### Inclusion Criteria

Adolescents who gave consent/assent and who understood the information and contents of the questionnaire on gambling were enrolled in the study.

### Exclusion Criteria

Adolescents who had known or previously diagnosed mental illness were excluded from the study.

### Study Instrument

The first Canadian problem gambling index prevalence survey took place in Quebec in 1991. 22 Subsequent studies were validated across New Brunswick, Alberta, Nova Scotia, Ontario, Manitoba, British Columbia, and Prince Edward Island. 22

The Problem Gambling Severity Index (PGSI), was validated by Natalie using a confirmatory factor analysis and Rasch modelling technique on a sample of over 25,000 gamblers compiled by merging data from the Canadian Community Health Survey and Canadian Problem Gambling Index (CPGI) integrated datasets. 23 Furthermore, Turner 24 et al reported a cross-validation of the Gambling Problem Severity Sub-scale of the Canadian Adolescent Gambling Index (CAGI/GPSS) in 2015 among adolescent students in grades 9–12 aged 13–20 years. The psychometric properties of the Canadian Problem Gambling Index (CPGI) was developed by Ferris and Wynne in 2001. The scale also reported a strong validity and psychometric properties. 25 It has nine variables with responses that range from 0=never; 1=sometimes; 2=most of the time and 3=always. The maximum obtainable score is 21 while the least score that could be obtained is zero. Respondents that obtained a total score of zero are regarded as having no problem gambling while a score of 1-2 indicates a low level of problems with few or no identified negative consequences. Also, a total score of 3-7 reveals a moderate level of problems leading to some negative consequences while a total score of 8 or more indicates problem gambling with negative consequences and a possible loss of control. Assessment of internet addiction was made using the internet addiction test. 26 Frangos 26 et al, validated this questionnaire in a meta-analysis of the reliability of Young's Internet addiction test. This is a validated questionnaire made up of 20 variables. Each variable has a 5-point Likert scale response including 0=not applicable; 1=rarely; 2=occasionally; 3=frequently; 4=often and 5=always. The maximum score that could be obtained by any respondent is 100 while the minimum score obtainable is zero. Total scores that range from 0-30 points are equivalent to a normal level of internet usage; scores of 31 to 49 are an indication of a mild level of internet addiction. Also, scores from 50 to 79 are regarded as a moderate level of internet use and scores of 80 to 100 reveal severe dependence on the internet.

### Sample size estimation

In other to attain a 95% confidence level and 5% precision for a population >100,000 a minimum sample size of 400 was estimated from the tables of sample sizes by Glenn 27 et al that would be necessary for given combinations of precision, confidence level, and variability for different population sizes. However, to increase the precision of the estimate, a sample size of 796 was used for the study.

### Ethical considerations

The Ethical clearance was obtained by the research and ethic committee of the Enugu State University Teaching Hospital with approval number ESUTP/C-MAC/RA/034/Vol.2/144. However, permission was sought and granted by the state school management board and the principal of the schools.

Informed consent was obtained from the subjects after describing to the participants, the objectives of the study. Assent was also obtained from children who are less than 18 years old.

### Social class estimation

Principal component analysis (PCA) using STATA statistical software version 12 was used to determine the socio-economic class of the family of the respondent. Input into the PCA included family ownership of household items including radio, television, refrigerator, car, bicycle and availability of electricity. Other variables included the type of residential building and type of toilet facility. For distribution cut points, quartiles were used. Each respondent was assigned the wealth index score of his/her family. The quartiles included Q1=poorest, Q2=the very poor, Q3=the poor and Q4, the least poor. The quartiles were then categorized into two groups, the low socio-economic class that included the poorest and very poor and high socio-economic class that included the poor and least poor groups.

### Problem gambling

This occurs when gambling becomes progressive and addictive with attendant psychological, physical, and social consequences. 22 Problem gambling is included in the American Psychiatric Association (APA's) Diagnostic and Statistical Manual, fifth edition (DSM-5) as an impulse-control disorder.

### Data analysis

Data entry and analysis were done using IBM Statistical Package for Social Sciences (SPSS) statistical software version 25. Continuous variables were represented using mean and standard deviation while categorical variables were summarized using frequencies and proportions. Correlation analysis, Chi-square test of statistical significance and multivariate analysis using binary logistic regression were used in the analysis. The level of statistical significance was determined by a p-value of <0.05.

In determining the predictors of problem gambling, variables that had a p-value of <0.2 after bivariate analysis were entered into the logistic regression model. The result of the logistic regression analysis was presented using an adjusted odds ratio and 95% confidence interval.

## Results

Table 1 shows the socio-demographic characteristics of the respondents. The mean age of the respondents was 15.6±1.8 years. The highest proportion of the respondents, 72.5% were in the age group 15-19 years while the least proportion, 2.6% were 20 years and above.

**Table 1:** Socio-demographic characteristics of respondents

Variable	Frequency (n=796)	Percent
**Age of respondents**		
Mean±(SD)	15.6±1.8	
**Age of respondents in groups**		
<15 years	198	24.9
15-19 years	577	72.5
≥20 years	21	2.6
**Gender**		
Male	384	48.2
Female	412	51.8
**Religion**		
Christianity	718	90.2
Islam	55	6.9
Traditional religion	23	2.9
**Educational attainment of Mother**		
No formal education	29	3.6
Primary education	168	21.1
Secondary education	247	31.0
Tertiary education	352	44.2
**Employment status of Father**		
Self-employed	580	72.9
Salaried employment	216	27.1
**Family socio-economic class**		
Poorest	213	26.8
Very poor	185	23.2
Poor	306	38.4
Least poor	92	11.6

The highest proportion of the respondents, 62.3% have never bet more than they could afford to lose in the last 12 months while the least proportion, 3.6% have done that almost always. Majority of the respondents, 70.4% have borrowed money or sold items to get money to gamble in the last 2 months while 11.1% do that sometimes. The highest proportion of the respondents, 60.2% never felt guilty about the way they gamble while 12.3% do that most of the time. Table 2

**Table 2:** Assessment of problem gambling in the last 12 months among the respondents

Variable	Never N (%)	Sometimes N (%) (n=796)	Most of the time N (%)	Almost always N (%)
In the last 12 months				
Have bet more than you could afford to lose.	496 (62.3)	202 (25.4)	69 (8.7)	29 (3.6)
Needed to gamble with larger amounts of money to get same excitement.	481 (57.9)	219 (27.5)	81 (10.2)	35 (4.4)
Gone back another day after gambling to win back lost money.	472 (59.3)	129 (16.2)	132 (16.6)	63 (7.9)
Borrowed money or sold items to get money to gamble.	560 (70.4)	88 (11.1)	63 (7.9)	85 (10.7)
Have felt you may have problem with gambling.	451 (56.7)	145 (18.2)	98 (12.3)	102 (12.8)
Gambling made you have health problems like stress and anxiety.	506 (63.6)	148 (18.6)	76 (9.5)	66 (8.3)
People have criticized your betting.	497 (62.4)	141 (17.7)	110 (13.8)	48 (6.0)
Gambling has caused financial problems for you or household.	530 (66.6)	118 (14.8)	85 (10.7)	63 (7.9)
Felt guilty about the way you gamble.	479 (60.2)	146 (18.3)	98 (12.3)	73 (9.2)

Table 3 shows the classification of problem gambling severity index among the respondents. The highest proportion of the respondents, 38.3% had problem gambling with negative consequences while the least proportion, 9.4% had low level of problem with few negative consequences.

**Table 3:** Classification of problem gambling severity index among the respondents

Variable	Frequency (n=796)	Percent (%)
**Problem gambling severity index**		
No problem gambling.	290	36.4
Low level of problem with few negative consequences.	75	9.4
Moderate level of problems with some negative consequences.	126	15.8
Problem gambling with negative consequences.	305	38.3

Table 4 shows the correlation matrix of variables. There is a weak positive correlation between gambling severity index and internet addiction. Increases in gambling severity index correlates with increases in internet addiction and this was found to be statistically significant, (n=796, r=0.254, p<0.001). There is a very weak negative correlation between gambling severity index and age in years. Increase in age correlates with decrease in gambling severity index but this was not found to be statistically significant, (n=796, r= -0.034, p=0.331).

**Table 4:** Correlation matrix of variables

Variable	(n=796) correlation co-efficient r, p value		
	Gambling severity index	Internet addiction	Age in years
Problem gambling severity index			
Internet addiction	r=0.254 p<0.001		
Age in years	r= -0.034 p=0.331	r=0.008 p=0.823	

Table 5 shows factors affecting gambling severity index among the respondents. The male respondents were 1.5 times more likely to have problem gambling when compared with the females, (AOR=1.5; (95%CI: 1.1-2.0). The respondents who have internet addiction were 3.5 times more likely to have problem gambling when compared with those who do not have an internet addiction, (AOR=3.5; 95%CI: 2.6-4.8).

**Table 5:** Factors affecting gambling severity index among the respondents

Variable	Problem gambling (n=796)		p value on bivariate analysis	**AOR (95% confidence interval) on multivariate analysis
	Yes N (%)	No N (%)		
**Age of respondents in groups**				
<15 years	136 (68.7)	62 (31.3)	0.016	0.3 (0.1 – 1.2)
15-19 years	352 (61.0)	225 (39.0)		0.3 (0.1 – 0.9)
≥20 years	18 (85.7)	3 (14.3)		1
**Gender**				
Male	263 (68.5)	121 (31.5)	0.005	1.5 (1.1 – 2.0)
Female	243 (59.0)	169 (41.0)		1
**Educational attainment of Mother**				
Tertiary education	206 (58.5)	146 (41.5)	0.008	0.7 (0.5 – 0.9)
Others*	300 (67.6)	144 (32.4)		1
**Employment status of Father**				
Self-employed	364 (62.8)	216 (37.2)	0.437	NA
Salaried employment	142 (65.7)	74 (34.3)		
**Family socio-economic class**				
Low socio-economic class	260 (65.3)	138 (34.7)	0.302	NA
High socio-economic class	246 (61.8)	152 (38.2)		
**Internet addiction**				
Yes	394 (73.2)	144 (26.9)	<0.001	3.5 (2.6 – 4.8)
No	112 (43.4)	146 (56.6)		1

* Secondary education and below NA Not applicable

** AOR Adjusted odds ratio

## Discussion

This study aims to determine the pattern and factors that predict gambling among secondary school adolescents. The prevalence of problem gambling among adolescents with negative consequences seen in this study was 38.3%. The prevalence of problem gambling obtained in this study is low when compared to world prevalence rates notwithstanding the differences in cut-offs and time frames in instruments used for assessments. For instance, a telephone survey conducted in the USA with a large sample of 2,274 U.S. adolescents aged 14–21 showed that 68% of the adolescents gambled in the past year while 11% gambled more than twice per week. 28 The prevalent rate of gambling seen in this study is lower than that seen in other African countries. For example, across Kenya, Ghana, Nigeria, Uganda, South Africa, and Tanzania, an average prevalent value of 54% had been documented. 29 Kenya had the highest prevalence rate of 76%, Uganda had 57%, while Ghana presented with the lowest prevalence in Africa (42%). 29

The high values obtained from the above studies compared to ours could be explained by the fact that gambling was not graded into severity patterns to ascertain those that have problem gambling with negative consequences. Moreover, the high prevalence in the African continent could be explained by the fact that the world's largest adolescent population is found in Africa. 30,31

The prevalence rates obtained in this study are higher than the prevalence rates of 0.2 -4.4 % and 12.3 %, 1.6% to 5.6% documented in Oceania, Brazil, Denmark and Albania, respectively. 32,33

Several reasons have been hazarded as causes of these varying prevalence rates in Africa when compared to other continents. Variations in the sample sizes as some authors used local and small sample sizes, while some used national samples, could account for these differences. 32

The study showed a high rate of gambling among adolescents from low socioeconomic status (SES), though this was not statistically significant. Studies have shown a lower rate of gambling in adolescents from low SES; however, an increased rate of problem gambling was noted among them when they start gambling at all. However, several studies 35 had shown that though adolescents from lower SES don't gamble much, on certain occasions while they are saddled with financial needs, they are more prone to gamble excessively. 35

Our study also showed that adolescents from parents who are not salary earners (i.e., not employed by the government) gamble more than those whose parents are government employed. It is documented that self-employment is associated with gambling frequency. 35

It is noted that males were more likely to gamble than their female counterparts. The male adolescents were 1.5 times more likely to have problem gambling when compared with the females. 4 A study has also documented that male adolescents were much higher than females on every measure and type of gambling. Though a study had shown a narrower gender gap in gambling, the study was mostly among the adult population. 36 It is hypothesized that female gambling tends to start in adulthood, while male involvement manifests in the adolescent period. A study 37 has suggested that the predictors for male preponderance for gambling were similar to that of other risky behaviours. This shows that there may be a similar etiology to gambling and other risky behaviours in adolescents. 37

This study showed increased prevalence of gambling with age. On further analysis using odd ratios and correlation matrix, our reportage showed that adolescents who were between 15 and 19 years were three times less likely to have problem gambling when compared to those who were 20 years and above. Welte et al 36 has also noted that gambling among adolescents increases with age. For instance, a prevalence of 9%,10%,11% and 19% were noted in adolescents aged 14-15,16-17 and,18-19 and 20-21% respectively. Hollen et al 38 with a large sample cohort of 3841 in the United Kingdom showed a high antecedent of gambling in the 17–24-year age group. The overall age prevalence was noted as 54% in those who were 17-year-old, 68% at 20 years and 66% at 24 years.

This study showed that increased age correlates with decrease in gambling severity index. Rahman et al 39 had noted associations between early age of onset with problem gambling severity indexed in their study. Furthermore, a recent study had also revealed a link between early age of gambling with at-risk/problem gambling among the adolescent internet gamblers. 40 Felsher et al 41 in their reportage on gambling behaviours among Canadian adolescents also noted a younger age of onset for gambling behaviours. This finding has also been corroborated by the submissions of Burge et al 42, Jimenez-Murcia et al 43 and Lynch et al 44. Risk-taking gestures at early adolescence may explain this finding. Besides, adolescence is a transition process marked with high impulsivity and vulnerability to addiction. 40

This study showed that adolescents who have internet addiction were 3.5 times more likely to have problem gambling when compared with those who do not have an internet addiction. Furthermore, it is noted in this study that increases in gambling severity index correlate with increases in internet addiction. Tsitsika ^45^ et al also noted that adolescents who are involved in gambling practices are more likely to have problematic internet use. ^45^

Several negative sequels such as fear of the unknown, indebtedness and guilt were documented in this study.

## Conclusion

There is an increased prevalence of gambling among adolescents. Adolescents who had problem gambling also had internet addiction. The male respondents were more likely to have problem gambling than their female folk.

## Data Availability

To protect the participants' anonymity, the data will not be shared.
